# The Effects of NT-1044, a Novel AMPK Activator, on Endometrial Cancer Cell Proliferation, Apoptosis, Cell Stress and *In Vivo* Tumor Growth

**DOI:** 10.3389/fonc.2021.690435

**Published:** 2021-08-05

**Authors:** Dario R. Roque, Lu Zhang, Weiya Z. Wysham, Jianjun Han, Wenchuan Sun, Yajie Yin, James N. Livingston, Ken W. Batchelor, Chunxiao Zhou, Victoria L. Bae-Jump

**Affiliations:** ^1^Division of Gynecologic Oncology, University of North Carolina at Chapel Hill, Chapel Hill, NC, United States; ^2^Department of Gynecologic Oncology, Shandong Cancer Hospital and Institute, Shandong First Medical University and Shandong Academy of Medical Sciences, Jinan, China; ^3^NovaTarg Therapeutics, First Flight Venture Center, Durham, NC, United States; ^4^Lineberger Comprehensive Cancer Center, University of North Carolina at Chapel Hill, Chapel Hill, NC, United States

**Keywords:** NT-1044, metformin, endometrial cancer, obesity, proliferation

## Abstract

**Objectives:**

Anti-diabetic biguanide drugs such as metformin may have anti-tumorigenic effects by behaving as AMPK activators and mTOR inhibitors. Metformin requires organic cation transporters (OCTs) for entry into cells, and NT-1044 is an AMPK activator designed to have greater affinity for two of these transporters, OCT1 and OCT3. We sought to compare the effects of NT-1044 on cell proliferation in human endometrial cancer (EC) cell lines and on tumor growth in an endometrioid EC mouse model.

**Methods:**

Cell proliferation was assessed in two EC cell lines, ECC-1 and Ishikawa, by MTT assay after exposure to NT-1044 for 72 hours of treatment. Apoptosis was analyzed by Annexin V-FITC and cleaved caspase 3 assays. Cell cycle progression was evaluated by Cellometer. Reactive oxygen species (ROS) were measured using DCFH-DA and JC-1 assays. For the *in vivo* studies, we utilized the *LKB1^fl/fl^p53^fl/fl^* mouse model of endometrioid endometrial cancer. The mice were treated with placebo or NT-1044 or metformin following tumor onset for 4 weeks.

**Results:**

NT-1044 and metformin significantly inhibited cell proliferation in a dose-dependent manner in both EC cell lines after 72 hours of exposure (IC50 218 μM for Ishikawa; 87 μM for ECC-1 cells). Treatment with NT-1044 resulted in G1 cell cycle arrest, induced apoptosis and increased ROS production in both cell lines. NT-1044 increased phosphorylation of AMPK and decreased phosphorylation of S6, a key downstream target of the mTOR pathway. Expression of the cell cycle proteins CDK4, CDK6 and cyclin D1 decreased in a dose-dependent fashion while cellular stress protein expression was induced in both cell lines. As compared to placebo, NT-1044 and metformin inhibited endometrial tumor growth in obese and lean *LKB1^fl/fl^p53^fl/fl^* mice.

**Conclusions:**

NT-1044 suppressed EC cell growth through G1 cell cycle arrest, induction of apoptosis and cellular stress, activation of AMPK and inhibition of the mTOR pathway. In addition, NT-1044 inhibited EC tumor growth *in vivo* under obese and lean conditions. More work is needed to determine if this novel biguanide will be beneficial in the treatment of women with EC, a disease strongly impacted by obesity and diabetes.

## Introduction

Endometrial cancer (EC) is known to be the most commonly diagnosed gynecologic malignancy in the United States (1). The incidence of EC is rising in tandem with the changing in hormonal factors and increasing prevalence of obesity and aging female population (2, 3). In 2021, it is estimated that 66,570 women will be diagnosed with an endometrial carcinoma, and an estimated 12,940 patients will succumb to this disease (1). Traditionally, EC has been subdivided into Type I and Type II tumors based on clinical and histologic differences (4–6). Type I tumors comprise 70-80% of endometrial adenocarcinomas and are thought to arise in part from unopposed estrogen stimulation. These tumors tend to be well-differentiated, of endometrioid histology, diagnosed at early stages, and associated with good prognosis. Obesity is predominantly associated with the development of type I endometrial cancer rather than type II endometrial cancer. Over 50% of endometrial cancers have been reported to attribute to overweight and obesity (2, 3). In addition, diabetes and insulin resistance have also been identified as independent risk factors for endometrial cancer and are associated with a 2-3 fold increased risk of developing this disease (7–11).

Metformin, a biguanide drug widely used for the treatment of Type II diabetes, has been shown to reduce cancer incidence and deaths among patients with diabetes (12–14). Metformin inhibits mitochondrial complex I in cells causing raised AMP/ATP ratios and, consequently leading to activation of AMPK (15). AMPK is the central regulator of energy utilization in cells, leading to increased glucose and fatty acid oxidation as well as other metabolic processes that generate ATP. The impact of these activities on metabolic disease is well documented (16); however, it is also clear that activation of AMPK leads to control of cell proliferation, in particular inhibition of the downstream PI3K/Akt/mTOR pathway (17–19). It should be noted that components of the PI3K/Akt/mTOR pathway are often mutated, amplified or aberrantly expressed in endometrial cancer (20–23). Activation of the PI3K/Akt/mTOR pathway, through *PIK3CA* amplifications, *PIK3CA*/*PIK3R1*/*PIK3R2* mutations and *PTEN* mutations/loss of function, has also been linked to more aggressive EC tumor behavior (21–24). Our laboratory has previously reported that metformin inhibits cell proliferation in a dose dependent manner in EC cell lines and decreased tumor growth in the *LKB1^fl/fl^p53^fl/fl^* mouse model of endometrioid endometrial cancer under obese and lean conditions (25, 26).

Metformin is currently being explored in the treatment of endometrial cancer in combination with hormonal and targeted therapies as well as chemotherapy; however, it should be noted that metformin did not improve on the efficacy of standard-of-care paclitaxel/carboplatin in advanced and recurrent endometrial cancer in NRG Oncology GOG286B (27), despite promising pre-clinical data in endometrial cancer cell lines and animal models (25, 26). Efforts are underway to develop new and improved pharmacologic versions of metformin for the treatment of diabetes mellitus, and it is logical that these same drugs may also have greater anti-tumorigenic benefits than metformin. Metformin is a highly basic molecule with a pKa of 11.5 and is present at <0.01% in its un-ionized form in blood, making metformin dependent on organic cation transporters (OCTs) for cellular entry (28). There are three OCTs, two of which (OCT1 and OCT3) are predominantly expressed on endometrial cancer cells; whereas, OCT2 is predominantly expressed in kidney and is responsible for metformin clearance in urine (29). This accounts for the short half-life of metformin (~1.7 hour), as well as the wide range of peak to trough drug levels, particularly in patients with impaired renal function (30). Therefore, novel biguanides with increased affinity for OCT1 and 3 and lower affinity for OCT2 could demonstrate higher tumor concentrations and a longer plasma half-life than metformin.

After screening approximately 140 biguanides, Novatarg has recently designed and synthesized NT-1044, a new biguanide with higher affinity for the OCT1 and OCT3 transporters. Although NT-1044 has 5.7 times greater affinity for OCT2 than metformin, affinity of NT-1044 for OCT1 and OCT3 is 153 times and 875 times greater than that of metformin, respectively, in HEK293 cells (**Table 1**). In the present study, we investigated the translational potential of NT-1044 as a therapeutic agent for endometrial cancer by evaluating the anti-tumor effects of this compound in endometrial cancer cell lines and a genetically engineered mouse model of endometrioid endometrial cancer.

**Table 1:** Potency of metformin *versus* NT-1044.

Compound	OCT Affinity IC_50_ (μM)		
	OCT1	OCT2	OCT3
Metformin	3532	7492	17500
NT-1044	213	1377	20

## Materials and Methods

### Cell Culture and Reagents

Two endometrial cancer cell lines, Ishikawa and ECC-1, and ES-T cell line (human endometrial stromal cells from a premenopausal woman, immortalized by hTERT) were utilized in this study. The ECC-1, Ishikawa and ES-T cells were grown in RPMI 1640 medium (11 mM glucose) with 5% fetal bovine serum, MEM medium (5.5 mM glucose) with 5% fetal bovine serum and L-glutamine, and DMEM/F12 with 10% fetal bovine serum under 5% CO2, respectively. All media included 100 units/ml penicillin and 100 microgram/ml streptomycin. NT-1044 was provided by NovaTarg Therapeutics (Research Triangle Park, NC). All primary and secondary antibodies were from Cell Signaling (Beverly, MA). Enhanced chemiluminescence reagents were bought from Amersham Inc (Arlington Heights, IL).

### Cell Proliferation Assays

The Ishikawa, ECC-1 and ES-T cells (4000 cells/well) were cultured in 96-well plates and exposed to various concentrations of metformin and NT1044 for 72 hours. Following treatments, 5 µl MTT solution (5 mg/ml) was added to each well, and the cells were cultured for an additional 1 hour. After aspiration of medium, 100 µl DMSO per well was used to terminate the reactions. The results were read by measuring absorption at 595 nm in a plate reader (Tecan, Cary, NC). The effect of NT-1044 or metformin on cell proliferation was calculated as a percentage of control cell growth obtained from DMSO treated cells grown in the same 96-well plates. Each experiment was performed in triplicate and repeated three times to assess for consistency of results.

### Cell Cycle Analysis

The ECC-1 and Ishikawa cells (2.5×10^5^ cells/ well) were cultured with or without NT1044 for 24 hours. The cells were subsequently collected by 0.05% Trypsin (Gibco), washed with PBS, and fixed in a 90% methanol solution. On the day of analysis, the cells were re-suspended in RNA A solution for 30 min at 37 °C, and then stained with PI staining solution for 10 min in the dark. Cell cycle progression was analyzed by Cellometer and analyzed by the FCS 4 Express Flow Cytometry Software (De Novo Software, Glendale, CA, USA). The experiments were repeated three times.

### Annexin V Staining Assay

Apoptotic cells were quantified by the Annexin-V FITC Kit (Biovison). Briefly, ECC-1 and Ishikawa cells were seeded into 6 well plates at 2.5 × 10^5^ cells/well overnight, and then the cells were treated with or without various concentrations of NT-1044 for 12 hours. The cells were harvested by 0.25% Trypsin and stained in 100 ul of Annexin-V and PI dual-stain solution for 15 min. The expression of Annexin V was detected by Cellometer, and analyzed by FCS 4 software. Apoptotic cells were expressed as a percentage of the total number of cells stained.

### Reactive Oxygen Species (ROS) Assay

Intracellular ROS production was detected using DCFH-DA assay. The ECC-1 and Ishikawa cells were plated in the appropriate media at 6000 cells/well in a 96-well plate overnight, and then treated with various concentrations of NT-1044 for 12 hours. 10 μl of 200 μM of DCFH-DA was added into each well and mixed gently. The fluorescence intensity was detected at Ex485/Em 485/530 nm by a Tecan plate reader. Each experiment was repeated at least three times for consistency of response.

### Cleaved Caspase 3 Assays

Caspase activity assays were performed with modifications as previously described (31). In brief, the ECC-1 and Ishikawa cells were plated in 6-well plates at a concentration of 2.5 × 10^5^ cells/well for 24 hours. The cells were treated with NT-1044 at different concentrations for 12 hours. 150-180 ul 1X caspase lysis buffer was added to each well. Protein concentration was determined using the BSA assay. 10-15 ug lysates in a black clear bottom 96-well plate were incubated with reaction buffer and 200 uM of cleaved caspase 3 substrates for 30 min. The fluorescence of each well was determined using a Tecan microplate reader. Each experiment was repeated three times to assess for consistency of results.

### Mitochondrial Membrane Potential Assay

Mitochondrial membrane potential was analyzed using the specific fluorescent probes JC-1 (32). The ECC-1 and Ishikawa cells were plated and treated with different concentrations of NT-1044 for 12 hours. Treated cells were then incubated with 2 uM JC-1 for 30 minutes at 37°C. The levels of the fluorescent probes were measured using a Tecan plate reader at two excitation/emission wavelength pairs. Each experiment was repeated three times to assess for consistency of results.

### Ethidium Bromide Competition Assay for OCT1-3 Affinity

HEK293 cells stably transfected with hOCT1, hOCT2, and hOCT3 vectors were cultured for 24-48 hours in 24-well plates, respectively. After medium was aspirated, Ethidium Bromide (EtBr) or EtBr plus NT-1044 diluted in HBSS was added to each well and cultured for 2.5 min at 37°C. The plate was washed with HBSS. Intracellular fluorescence was determined by a plate reader (Tecan) at 535/590-nm excitation and emission wavelengths. Nonspecific fluorescence in control cells was subtracted from fluorescence in hOCT-overexpressing cells to yield data representing hOCT-specific fluorescence (33).

### Western Immunoblotting

The Ishikawa and ECC-1 cells were seeded at 2.5 × 10^5^ cells/well in 6-well plates and then treated with various concentrations of NT-1044 for 24 hours. The treated cells were lysed in an RIPA buffer (1% NP40, 0.5 sodium deoxycholate and 0.1% SDS) and centrifuged at 12,000 rpm for 15 min to collect supernatants. The protein concentration was determined using the BCA assay kit (Thermo Fisher Scientific). Equal amounts of protein were separated by SDS-PAGE gels and transferred to a PVDF membrane for 2 hours. The membrane was blocked by 5% milk and then incubated with the following primary antibodies: phos-AMPK and S6, MCL-1, BCL-XL, CDK4, CDK6, Bip, PERK and cyclin D1 at 4°C overnight. The membrane was washed with TBS-T and incubated with anti-rabbit or anti-mouse secondary antibodies for 1 hour. Enhanced chemiluminescence reagents was used to visualize protein binding through a gel imaging analysis system (Bio-Rad). The band densitometry was performed using Image J software. The relative expression of target protein was normalized to the expression of β-actin or α-Tubulin. Each experiment was repeated three times to assess for consistency of results.

### NT-1044 Treatment in a Transgenic Mouse Model of Endometrial Cancer

For our *in vivo* studies, we utilized an *LKB1^fl/fl^p53^fl/fl^* genetically engineered mouse model. This model was developed in our lab, and these mice develop endometrioid histology endometrial cancer (25). For this model, LKB1 and p53 are somatically inactivated *via* injection of AdCre virus directly into one uterine horn of the mice. The mice develop invasive endometrial cancer approximately 8 weeks after AdCre virus injection. Given that obesity and insulin resistance are known risk factors for the development of endometrial cancer, we assessed NT-1044 and metformin treatment in both obese and lean mice. To accomplish this, mice were fed either a high fat diet (HFD, 60% calories, obese group) or low fat diet (LFD, 10% calories, lean) starting 3 weeks after birth. At 6 weeks after birth, AdCre was injected into the right uterine horn of all mice. Eight weeks after AdCre injection, we treated the mice with either NT-1044 (200mg/kg/day, oral garage, daily) or metformin (200mg/kg, drinking water, daily) for a duration of 4 weeks in HFD and LFD groups (n = 15/group). Body weight and blood glucose were monitored weekly during the treatment. All mice were euthanized at the end of treatment. Tumor tissue and blood samples were collected. Animal experiments were approved by our Institutional Animal Care and Usage Committee in accordance with NIH guidelines.

### Immunohistochemical Analysis

The mouse tumor tissue was formalin-fixed and paraffin-embedded. Slides (5 μm) were first incubated with protein block solution (Dako) for 1 hour and then with the primary antibodies for Ki-67 (1:400), phosphorylated-S6 (1:300) and phosphorylated-AMPK (1:100) for 2 hours at room temperature. The slides were then washed and incubated with appropriate secondary antibodies at room temperature for 1 hour. The slides were washed, and the specific staining was visualized using the Signal Stain Boost Immunohistochemical Detection Reagent (Cell Signaling Technology), according to the manufacturer’s instructions. Individual slides were scanned using the Aperio™ ScanScope (Aperio Technologies, Vista, CA), and digital images were analyzed for target protein expression using Aperio™.

### Primary Culture

A normal endometrium of proliferative phase (36 years old) was collected in the operating room of the Department of Gynecologic Oncology, Shandong Cancer Hospital and institute, China. A specific written informed consent was obtained from patient, and the study was approved by the Institutional Ethics Committee of Shandong Cancer Hospital and Institute. Tissue was then digested in 0.2% collagenase IA for 45 min hours at 37°C water bath with shaking. Epithelial and stromal cells were separated using number 100 and number 400 sieves. 8000 cells/well were seeded into 96-well plated and cell proliferation was measured with MTT assay 72 hours after treatment.

### Statistical Analysis

All experiments were repeated a minimum of three times. Results for experiments were normalized to the mean of the control and analyzed using the Student t-test using GraphPad software (La Jolla, CA, USA). Differences were considered significant if the p value was less than 0.05 (p<0.05) with a confidence interval of 95%.

## Results

### Effect of NT-1044 on Endometrial Cancer Cell Proliferation

**Figure 1:**
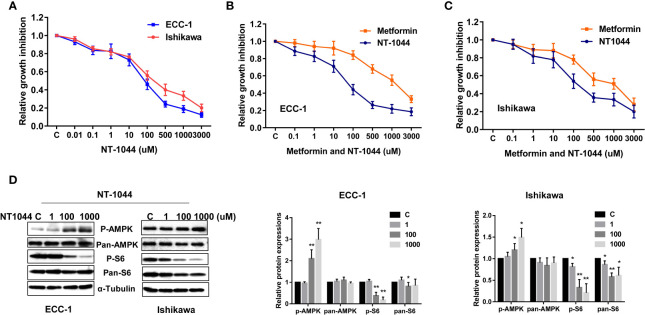
NT-1044 inhibited cell proliferation and activated AMPK pathway in EC cells. The ECC-1 and Ishikawa cells were cultured in regular media for 24 hours, and then treated with the indicated concentrations of NT-1044 and metformin in 96-well plates for 72 hours. Cell proliferation was assessed by MTT assay. NT-1044 and metformin significant inhibited cell proliferation in a dose dependent manner **(A–C)**. IC50 value of NT-1044 for ECC-1 = 87 μM; Ishikawa=218 μM. The ECC-1 and Ishikawa cells were treated with NT-1044 for 18 hours. Western blotting showed that NT-1044 increased phosphorylation of AMPK expression and decreased phosphorylation of S6 expression **(D)**. The results are shown as the mean ± SE of triplicate samples and are representative of three independent experiments. *p < 0.05, **p < 0.01.

The affinity of NT-1044 and metformin for OCT1-3 were compared at different concentrations using spectrophotometer in HEK293 cells. The results showed that the affinity of NT-1044 to OCT1 and OCT3 is stronger than that of metformin (**Table 1**). Next, we examined the effect of NT-1044 and metformin on cell proliferation in the ECC-1 and Ishikawa endometrial cancer cell lines. The cells were incubated for 72 hours with varying concentrations of NT-1044 and metformin and cellular viability was analyzed with the MTT assay. The results from the MTT assay showed a progressive decrease in cell proliferation with successive increases in the concentrations of NT-1044 and metformin (**Figure 1A**). NT-1044 exhibits dose-dependent effects on cellular growth for both cell lines at significantly lower doses than that of metformin. As shown in **Figures 1B, C**, the mean IC_50_ value of NT-1044 was approximately 87 ± 22.49 μM and 218 ± 50.76 μM for the ECC-1 and Ishikawa cells; the mean IC50 values of metformin were 1330 ± 211.38 uM for Ishikawa and 2750 ± 173.31 uM for ECC-1, respectively. The results indicated that the both cell lines were sensitive to NT-1044 treatment and that NT-1044 had a greater effect on inhibiting cell proliferation than metformin.

In order to determine whether NT1044 and metformin alter cell proliferation of normal endometrial cells, Normal TERT-immortalized endometrial stromal cell line, ES-T, and a primary culture of normal endometrium (proliferative phase) were used. ES-T, primary culture of epithelial cells and stromal cells were treated with either metformin, NT1044 or vehicle control. Metformin led to a modest growth inhibition in ES-T and primary culture cells after 72 hours of treatment, while NT1044 reduced cell proliferation with an IC50 of 688 uM in ES-T cells (**Supplemental Figure 1**). NT1044 exhibits greater effect on inhibiting cell proliferation than metformin in normal endometrial cells. These results indicate that normal endometrial cells are relatively metformin and NT1044 resistant compared to ECC-1 and Ishikawa cells.

Given that metformin exhibits anti-tumorigenic effects by activating AMPK and inhibiting mTOR pathways, we examined expression of key targets of these pathways after treatment with NT-1044. As illustrated in **Figure 1D**, western immunoblotting showed that NT-1044 significantly increased expression of AMPK phosphorylation and dramatically decreased the expression of phosphorylated S6 in both cell lines after 18 hours of treatment with NT-1044. Expression of pan-AMPK was not affected by NT-1044. However, NT1044 reduced the expression of pan-S6 in Ishikawa cells. These results indicate that NT-1044 inhibits cell proliferation *via* activation of AMPK and subsequent decreased phosphorylation of the S6 protein, resulting in inhibition of the mTOR pathway.

### Effect of NT-1044 on Cell Cycle

**Figure 2:**
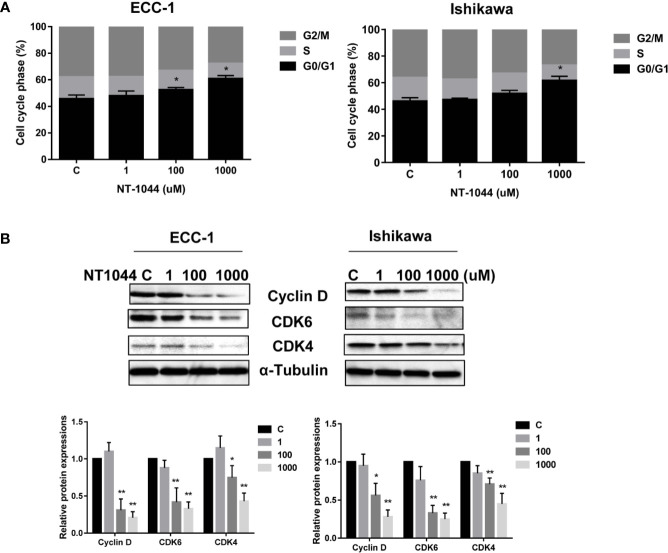
NT-1044 induced cell cycle G1 arrest in EC cells. The ECC-1 and Ishikawa cells were treated with NT-1044 for 24 hours. Cell cycle progression was assessed by Cellometer. NT-1044 induced cell cycle G1 arrest in a dose-dependent manner in both cell lines **(A)**. Western blotting showed that NT-1044 decreased the expression of CDK4, CDK6 and cyclin D in a dose-dependent manner in both cell lines after 24 hours of exposure **(B)**. *p < 0.05, **p < 0.01.

After treating the ECC-1 and Ishikawa cells with NT-1044, the cell cycle profile was analyzed to evaluate the underlying mechanism of growth inhibition by NT-1044. As illustrated in **Figure 2A**, NT-1044 (1-1000 uM) resulted in dose dependent G1 cell cycle arrest in the ECC-1 and Ishikawa cell lines. NT-1044 at a dose of 1 mM increased the G1 population from 47.1 ± 2.72% to 63.8% ± 2.28 in the ECC-1 cells, and from 46.1 ± 3.06% to 57.3 ± 2.67% in the Ishikawa cells, respectively, compared with control groups (p<0.01). The synthesis of cyclin D1 is initiated during G1 and, through interaction with the cyclin-dependent kinases (CDK) 4 and 6, drives the G1/S phase transition (34). The expression of CDK4, CDK6 and Cyclin D1 were measured by Western blotting. Treatment of the ECC-1 and Ishikawa cells with NT-1044 for 24 hours led to a dose dependent decrease in Cyclin-D1, CDK6 and CDK-4 (**Figure 2B**). These results are basically consistent with our previous research on the effect of metformin on the cell cycle (25, 26).

### Effect of NT-1044 on Apoptosis

**Figure 3:**
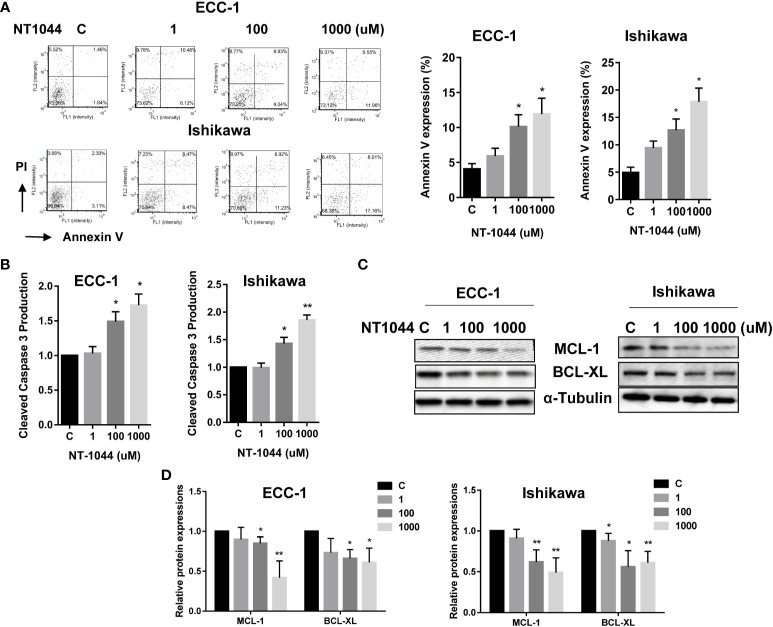
NT-1044 induced apoptosis in EC cells. The ECC-1 and Ishikawa cells were cultured for 24 hours and then treated with NT-1044 for 12 hours. NT-1044 increased Annexin V expression in a dose-dependent manner in both cells **(A)**. Both cell lines were treated with NT-1044 for 12 hours. Cleaved caspase 3 was measured by ELISA assay. The results showed that NT-1044 increased cleaved caspase 3 activity in both cell lines **(B)**. Western blotting showed that NT-1044 decreased the expression of BCL-XL and MCL-1 in a dose-dependent manner in both cell lines after 18 hours of exposure **(C, D)**. *p < 0.05, **p < 0.01.

The effect of NT-1044 on apoptosis was evaluated by Annexin V and cleaved caspase 3 assays in both cell lines. Treatment of both cell lines with different concentrations of NT-1044 for 12 hours significantly increased Annexin V expression in a dose dependent manner as demonstrated in **Figure 3A** (p<0.01). Following incubation with 1 mM NT-1044, Annexin V-positive cells were increased from 4.2% to 13.4% for ECC-1 cells and 5.1% to 17.5% for Ishikawa cells, respectively. Treatment of both cell lines with 1 mM NT-1044 for 12 hours increased cleaved caspase 3 activity by approximately 70-85% compared with the untreated groups (**Figure 3B**, p<0.01). Western blotting analysis found that NT-1044 reduced expression of the anti-apoptotic proteins BCL-XL and MCL-1 in a dose-dependent manner after treatment for 18 hours (**Figures 3C, D**). These results suggest that NT-1044 inhibits endometrial cancer cell growth through the induction of apoptosis and cell cycle G1 arrest.

### NT-1044 Induces Cellular Stress

**Figure 4:**
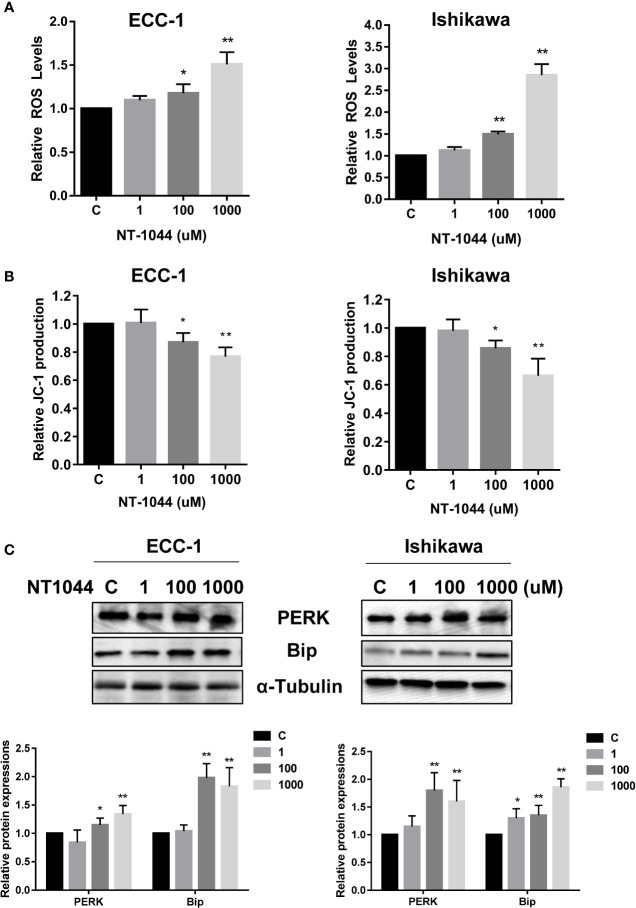
NT-1044 induced cell stress in EC cells. The ECC-1 and Ishikawa cells were cultured for 24 hours and then treated with NT-1044 for 12 hours. Reactive Oxygen Species (ROS) was detected using the DCFH-DA Assay. Mitochondrial membrane potential was measured by JC-1 assay. NT-1044 significantly increased ROS products **(A)** and decreased JC-1 levels **(B)** in a dose dependent manner in the both cells. Both cells were treated with NT-1044 for 24 hours. Western blotting showed that NT-1044 increased the expression of BIP and PERK in the both cells **(C)**. *p < 0.05, **p < 0.01.

Given that metformin activates cellular stress in many types of solid cancers, we examined the effects of NT-1044 on cellular stress and mitochondrial membrane potential using DCFH-DA and JC-1 assays, respectively. After a 12-hour treatment with varying concentrations of NT-1044, a dose-dependent response on reactive oxygen species (ROS) assays can be seen, illustrating the effects of NT-1044 on inducing intracellular stress (**Figure 4A**). ROS was increased by 45-165% from baseline in ECC-1 and Ishikawa cells at a dose of 1 mM NT-1044. Meanwhile, **Figure 3B** shows significantly decreased mitochondrial membrane potential of 24.1-33.7% in ECC-1 and Ishikawa cells after treatment with NT-1044 for 12 hours at 1 mM (**Figure 4B**). We used western immunoblotting to analyze the changes in protein kinase-like endoplasmic reticulum kinase (PERK) and binding immunoglobulin protein (Bip), two proteins related to cell stress, after the cells were treated with different concentrations of NT-1044 for 24  hours. The data showed that the expression of PERK and Bip increased with increasing doses of NT-1044 (**Figure 4C**). These results indicate that NT-1044 induces intracellular oxidative stress in EC cells.

### NT-1044 Inhibits Tumor Growth in the *LKB1 ^fl/fl^p53^fl/fl^* Mouse Model of Endometrial Cancer

**Figure 5:**
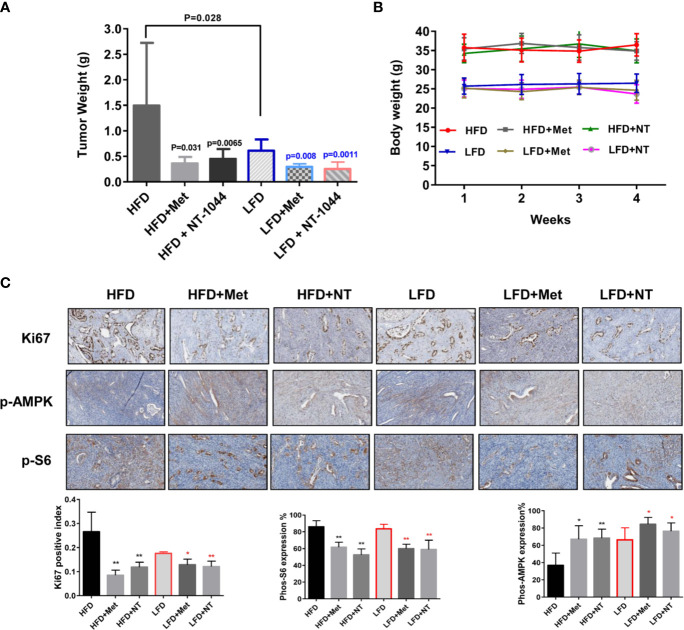
NT-1044 inhibited endometrial tumor growth in both the obese and lean *LKB1^fl/fl^p53^fl/fl^* mice. *LKB1^fl/fl^ p53^fl/fl^* mice were fed high fat diet (HFD) or low fat diet (LFD) at 3 weeks of age to induce obesity. The mice were divided into six groups: obese, obese + metformin, obese+NT-1044, Lean, lean + metformin. Lean+NT-1044. The obese and lean mice in both groups were treated with NT-1044 or metformin (200 mg/kg, oral gavage for NT1044 and drinking water for metformin) or placebo for 4 weeks. Obesity promoted tumor growth in obese mice versus lean mice. Either metformin or NT-1044 significantly reduced tumor weight in the obese and lean mice, with a greater impact on tumor weight in obese mice **(A)**. During treatment, there were no significant changes in body weight in the six groups **(B)**. Change in expression of Ki-67, phosphorylated-S6 and phosphorylated-AMPK were assessed by immunohistochemistry in the endometrial cancer tissues. The expression of Ki-67 and phosphorylated-S6 was reduced and phosphorylated-AMPK was increased in both groups after NT-1044 or metformin treatment **(C)**. *p < 0.05, **p < 0.01.

To validate and compare the anti-tumorigenic potential of NT-1044 and metformin *in vivo* under obese and lean conditions, we fed *LKB1^fl/fl^p53^fl/fl^* mice with either a HFD (obese) at 3 weeks of age to induce obesity or fed them a LFD as lean controls. The obese and lean mice were treated with either placebo, NT-1044 or metformin at a dose of 200 mg/kg/day for 4 weeks (15 mice/per group). During treatments, tumor growth was monitored by palpation twice a week. Regular twice-weekly measurements yielded no changes in random blood glucose and body weight during NT-1044, metformin and placebo treatments. All mice were evidently well tolerated during the treatments. Obesity accelerated tumor growth with a 2.1-fold increase in tumor weight at sacrifice compared to mice fed a LFD. Both obese and lean mice treated with metformin had a significant reduction in tumor weight (**Figures 5A, B**, p<0.05). NT-1044 exhibited similar anti-tumor effects in obese and lean mice compared with metformin. However, metformin and NT-1044 had a more pronounced impact on the tumor growth of obese mice (70.3 and 75.8% reduction in tumor weight, respectively) compared to 58.1 and 63.8% reduction in tumor weight with NT-1044 and metformin treatment in lean mice, respectively (p<0.05). Although NT-1044 was significantly more potent than metformin for its *in vitro* anti-tumor activity, there was not a difference between NT-1044 and metformin in reduction of tumor weight under obese and lean conditions. The possible reasons are the duration of treatment and large doses of both agents used that did not allow the detection of a difference in drug potency in the *LKB1^fl/fl^p53^fl/fl^* mice (35, 36). Overall, these results suggest that obesity promotes endometrial tumor growth and NT-1044 and metformin effectively suppresses the endometrial tumor growth in both obese and lean mice, with greater anti-tumorigenic effects in the setting of obesity.

To further investigate the anti-tumorigenic mechanism of NT-1044 *in vivo*, the expression of Ki-67, phosphorylated S6 and phosphorylated AMPK in the endometrial tumor tissues was evaluated by immunohistochemistry (**Figure 5C**). As expected, the expression of the proliferation marker Ki-67 was significantly reduced in the endometrial tumors following metformin and NT-1044 treatments, quantified as 55.1-67% decrease in tumors of obese mice and 27.2-31.4% in tumors of lean mice compared to controls (p<0.05). Consistent with our results *in vitro*, NT-1044 and metformin significantly reduced the expression of phosphorylated S6 and increased the expression of phosphorylated AMPK in obese and lean mice compared with the untreated mice, suggesting that metformin and NT1044 inhibited tumor growth through the AMPK/mTORC1 pathway *in vivo* (p<0.05). Together, these results further confirm that NT-1044 and metformin inhibits tumor growth of EC, potentially *via* targeting of the AMPK/mTOR/S6 pathway *in vivo* in both obese and lean mice.

## Discussion

The mechanism of the inhibitory effect of metformin on proliferation of cancer cells and tumor growth has been suggested to be associated with cell cycle arrest, promotion of apoptosis, inhibition of metastasis and induction of cellular stress through the AMPK/mTOR pathway (37, 38). In this study, we sought to investigate the effects of NT-1044, an AMPK activator, on cell proliferation in two human endometrial cancer cell lines and on tumor growth in an endometrial cancer mouse model. When compared to metformin, NT-1044 shows more potent anti-proliferative activity than metformin in EC cells and the similar anti-tumor activities in the *LKB1^fl/fl^ p53^fl/fl^* mouse model of EC. In addition, we found that NT-1044 suppressed cell growth, induced apoptosis and G1 cell cycle arrest, caused cellular stress, activated AMPK and inhibited mTOR pathways *in vitro and in vivo*.

Metformin inhibits the proliferation of cancer cells, including EC cells, by inhibiting cell cycle progression in the G1 phase and/or G2 phase through the downregulation of cyclins, and cyclin dependent kinases (38, 39). Our previous work confirmed that metformin significantly induced cell cycle arrest in G1 phase in the Ishikawa and ECC-1 cells (25, 26). Similarly, we found that NT-1044 promoted cell cycle-G1 arrest of ECC-1 and Ishikawa cells in a dose-dependent fashion in the current study. This effect was accompanied by decreases in the Cyclin-D1, CDK-6 and CDK-4 proteins, all of which are involved in cell cycle progression. These results confirm that NT-1044 has similar effects to metformin on cell cycle progression in EC.

Exposure to metformin has been shown to increase apoptosis in different types of cancers including EC cells, culminating in decreased cell viability (18, 26, 40). Enhanced apoptosis was reported to be mediated by increased caspase-3 activation and PARP cleavage (37). In our previous work, metformin induced caspase 3 activity but only at high concentrations of treatment (2-5 mM) (26). Lower doses of metformin had little effect on caspase-3 activity. Similar results have demonstrated that 10 mM metformin significantly increased the proportion of Annexin V positive cells and the expression of cleaved caspase-3 in Ishikawa cells (39). Whether metformin induces apoptosis is controversial as it has also failed to induce apoptosis in prostate and breast cancer cell lines at similar doses of treatment. The underlying reason for this difference may be attributed to the duration and concentration of metformin treatment as well as the status of wild type p53 (37, 41). Metformin may directly affect the expression of p53 in sensitive cancer cells, which in turn leads to regulation of p53 downstream targets and to induction of apoptosis (42). Given that metformin has poor lipophilicity and its bio-distribution in cancer cells relies on OCT1, OCT2 and OCT3, it is also possible that induction of apoptosis is related to OCT1/3 receptor expression and the biguanide potency. NT-1044 has significantly higher affinity for both OCT1 (IC50 213 μM vs. 3,532 μM) and OCT3 (IC50 20 μM vs. 17,500 μM) when compared to metformin (**Table 1**). Our preliminary studies on transporter proteins have shown that OCT1 and OCT3 are present in both endometrial cancer cell lines and endometrial tumors (**Supplemental Figure 2**). The higher potency of NT-1044 as well as the higher affinity to the OCT1 and OCT3 transporter when compared to metformin, may explain why NT-1044 is more effective at inducing apoptosis in our current study. However, additional research is needed to determine if OCT1 and OCT3 expression is in fact a predictor of treatment response to biguanides including NT-1044.

Inhibition of cell proliferation by NT-1044 was accompanied by inhibition of the AMPK/mTOR pathway. AMPK/mTOR pathway is well known to be regulated cancer cell growth and survival. The activation of AMPK reduces cell proliferation through negatively regulates mTOR signaling (43). Recent results showed that NT-1044 exhibited higher AMPK activation than metformin at the same dosage in differentiated human adipocytes (44).In its role as an AMPK activator, the anti-tumorigenic activity of NT-1044 appears similar to that of metformin, which has been shown to significantly decrease proliferation of several human cancer cell lines *in vitro*, including endometrial cancer (25, 26, 45–47). Since AMPK negatively controls mTOR activation and mTOR is a downstream effector of AKT, Activation of AMPK is considered to be a possible therapeutic target for cancers that contain high AKT activity (48). Given type I endometrial cancers exhibit a high frequency of PTEN deletions and mutations, leading to the activation of AKT, AMPK activation may have therapeutic potential for this disease (49). In this study, ECC-1 and Ishikawa cells have low expression of wild PTEN, but RL-95-2 cells, a PTEN mutant EC cell line, exhibited the similar sensitivity to metformin and NT1044 compared to ECC-1 and Ishikawa cells, suggesting that PTEN status may not play a big role in inhibition of cell growth in NT1044-treated endometrial cancer cells. Several studies have confirmed that loss of PTEN significantly enhances the sensitivity to rapamycin, an mTOR inhibitor (50–52). The results of the present study suggested that NT1044 may be promising therapy for type I endometrial cancer, which more frequently have PTEN mutations, as well as type II endometrial cancers, which less frequently have PTEN mutations.

Lastly, we explored the activity of NT-1044 *in vivo* in an endometrial cancer mouse model. We also assessed whether this drug would have different activity in lean versus obese mice given that obesity has been linked to both an increased risk of developing endometrial cancer and an increased risk of mortality from the disease (53, 54). In the obese mice, NT-1044 inhibited tumor growth by 76% (p=0.0065) whereas in the lean mice, it inhibited tumor growth by 64% (p=0.0011), suggesting that NT-1044 is more effective in the setting of obesity. NT-1044 exhibited similar anti-tumor activity compared to metformin. These findings are not surprising given the association between obesity, insulin resistance and endometrial cancer. In a pre-operative window study, metformin was shown to have increased activity against endometrial cancer in the subset of patients with metabolomic profiles consistent with increased insulin resistance (55).

## Conclusion

As our work has demonstrated, NT-1044 has greater potency than metformin *in vitro* and comparable effects to metformin *in vivo* under obese and lean conditions. In addition, we recently found that NT1044 inhibited cell proliferation in normal endometrial stromal and epithelial cells, suggesting that NT1044 may have potential to treat non-malignant hyperplastic diseases in uterus (**Supplemental Figure 1**). In pharmacologic studies of NT-1044 for diabetes treatment, NT-1044 demonstrated the anticipated pharmacologic activity of a biguanide but at just 1/5 of the metformin dose (data not shown). Therefore, NT-1044 represents a novel biguanide with improved pharmacologic features over metformin that include increased selectivity of transporters and a longer plasma half-life, although this did not result in an increased benefit in its anti-tumorigenic effects in the *LKB1 ^fl/fl^p53^fl/fl^*. Thus, future work will focus on expansion of the NT-1044 treatment studies into additional patient-derived xenograft mouse models of endometrial cancer of varying genomic backgrounds to assess for potential biomarkers of NT-1044 response that may also align with obesity status, in particular mutations that affect the AMPK/mTOR pathway, a pathway well-known to be altered in both obesity and endometrial cancer.

## Data Availability Statement 

The original contributions presented in the study are included in the article/**Supplementary Material**. Further inquiries can be directed to the corresponding authors.

## Ethics Statement

The animal study was reviewed and approved by Institutional Animal Care and Use Committee, UNC.

## Author Contributions

DR, LZ, WW, JH, and WS performed the experiments *in vitro*. LZ, JH, and YY performed animal experiments. DR, LZ, and WW participated in analyzing and interpreting the data. DR and CZ wrote the manuscript. JL and KB provided NT-1044. CZ and VB-J designed experiments, revised the manuscript and provided financial support. All authors contributed to the article and approved the submitted version.

## Funding

This work is supported by: (1) VB-J: American Cancer Society (ACS) Research Scholar Grant - RSG CCE 128826. (2) VB-J: NIH/NCI - R37CA226969.

## Conflict of Interest

JL and KB are employees and shareholders of NovaTarg Therapeutics.

The remaining authors declare that the research was conducted in the absence of any commercial or financial relationships that could be construed as a potential conflict of interest.

## Publisher’s Note

All claims expressed in this article are solely those of the authors and do not necessarily represent those of their affiliated organizations, or those of the publisher, the editors and the reviewers. Any product that may be evaluated in this article, or claim that may be made by its manufacturer, is not guaranteed or endorsed by the publisher.

## References

[1] Siegel RL Miller KD Fuchs HE Jemal A. Cancer Statistics, 2021. CA Cancer J Clin (2021) 71:7–33. 10.3322/caac.21654 33433946

[2] Fader AN Arriba LN Frasure HE von Gruenigen VE. Endometrial Cancer and Obesity: Epidemiology, Biomarkers, Prevention and Survivorship. Gynecol Oncol (2009) 114:121–7. 10.1016/j.ygyno.2009.03.039 19406460

[3] von Gruenigen VE Gil KM Frasure HE Jenison EL Hopkins MP. The Impact of Obesity and Age on Quality of Life in Gynecologic Surgery. Am J Obstet Gynecol (2005) 193:1369–75. 10.1016/j.ajog.2005.03.038 16202728

[4] Prat J Gallardo A Cuatrecasas M Catasus L. Endometrial Carcinoma: Pathology and Genetics. Pathology (2007) 39:72–87. 10.1080/00313020601136153 17365824

[5] lHecht JL Mutter GL. Molecular and Pathologic Aspects of Endometrial Carcinogenesis. J Clin Oncol (2006) 24:4783–91. 10.1200/JCO.2006.06.7173 17028294

[6] Sherman ME. Theories of Endometrial Carcinogenesis: A Multidisciplinary Approach. Mod Pathol (2000) 13:295–308. 10.1038/modpathol.3880051 10757340

[7] Friberg E Mantzoros CS Wolk A. Diabetes and Risk of Endometrial Cancer: A Population-Based Prospective Cohort Study. Cancer Epidemiol Biomarkers Prev (2007) 16:276–80. 10.1158/1055-9965.EPI-06-0751 17301260

[8] Friberg E Orsini N Mantzoros CS Wolk A. Diabetes Mellitus and Risk of Endometrial Cancer: A Meta-Analysis. Diabetologia (2007) 50:1365–74. 10.1007/s00125-007-0681-5 17476474

[9] Soliman PT Wu D Tortolero-Luna G Schmeler KM Slomovitz BM Bray MS. Association Between Adiponectin, Insulin Resistance, and Endometrial Cancer. Cancer (2006) 106:2376–81. 10.1002/cncr.21866 16639730

[10] Chia VM Newcomb PA Trentham-Dietz A Hampton JM. Obesity, Diabetes, and Other Factors in Relation to Survival After Endometrial Cancer Diagnosis. Int J Gynecol Cancer (2007) 17:441–6. 10.1111/j.1525-1438.2007.00790.x 17362320

[11] Cust AE Kaaks R Friedenreich C Bonnet F Laville M Lukanova A. Plasma Adiponectin Levels and Endometrial Cancer Risk in Pre- and Postmenopausal Women. J Clin Endocrinol Metab (2007) 92:255–63. 10.1210/jc.2006-1371 17062769

[12] Evans JM Donnelly LA Emslie-Smith AM Alessi DR Morris AD. Metformin and Reduced Risk of Cancer in Diabetic Patients. Bmj (2005) 330:1304–5. 10.1136/bmj.38415.708634.F7 PMC558205 15849206

[13] Libby G Donnelly LA Donnan PT Alessi DR Morris AD Evans JM. New Users of Metformin Are at Low Risk of Incident Cancer: A Cohort Study Among People With Type 2 Diabetes. Diabetes Care (2009) 32:1620–5. 10.2337/dc08-2175 PMC2732153 19564453

[14] Bowker SL Majumdar SR Veugelers P Johnson JA. Increased Cancer-Related Mortality for Patients With Type 2 Diabetes Who Use Sulfonylureas or Insulin: Response to Farooki and Schneider. Diabetes Care (2006) 29:1990–1. 10.2337/dc06-0997 16873829

[15] Hinke SA Martens GA Cai Y Finsi J Heimberg H Pipeleers D. Methyl Succinate Antagonises Biguanide-Induced AMPK-Activation and Death of Pancreatic Beta-Cells Through Restoration of Mitochondrial Electron Transfer. Br J Pharmacol (2007) 150:1031–43. 10.1038/sj.bjp.0707189 PMC2013909 17339833

[16] Hawley SA Gadalla AE Olsen GS Hardie DG. The Antidiabetic Drug Metformin Activates the AMP-Activated Protein Kinase Cascade *via* an Adenine Nucleotide-Independent Mechanism. Diabetes (2002) 51:2420–5. 10.2337/diabetes.51.8.2420 12145153

[17] Gallagher EJ LeRoith D. Epidemiology and Molecular Mechanisms Tying Obesity, Diabetes, and the Metabolic Syndrome With Cancer. Diabetes Care (2013) 36 Suppl 2:S233–9. 10.2337/dcS13-2001 PMC3920794 23882051

[18] Quinn BJ Kitagawa H Memmott RM Gills JJ Dennis PA. Repositioning Metformin for Cancer Prevention and Treatment. Trends Endocrinol Metab (2013) 24:469–80. 10.1016/j.tem.2013.05.004 23773243

[19] Noto H Goto A Tsujimoto T Noda M. Cancer Risk in Diabetic Patients Treated With Metformin: A Systematic Review and Meta-Analysis. PloS One (2012) 7:e33411. 10.1371/journal.pone.0033411 22448244 PMC3308971

[20] Gehrig PA Bae-Jump VL. Promising Novel Therapies for the Treatment of Endometrial Cancer. Gynecol Oncol (2010) 116:187–94. 10.1016/j.ygyno.2009.10.041 PMC4103663 19903572

[21] Dedes KJ Wetterskog D Ashworth A Kaye SB Reis-Filho JS. Emerging Therapeutic Targets in Endometrial Cancer. Nat Rev Clin Oncol (2011) 8:261–71. 10.1038/nrclinonc.2010.216 21221135

[22] Cheung LW Hennessy BT Li J Yu S Myers AP Djordjevic B. High Frequency of PIK3R1 and PIK3R2 Mutations in Endometrial Cancer Elucidates a Novel Mechanism for Regulation of PTEN Protein Stability. Cancer Discovery (2011) 1:170–85. 10.1158/2159-8290.CD-11-0039 PMC3187555 21984976

[23] Kandoth C Schultz N Cherniack AD Akbani R Liu Y Shen H. Integrated Genomic Characterization of Endometrial Carcinoma. Nature (2013) 497:67–73. 10.1038/nature12113 23636398 PMC3704730

[24] Salvesen HB Carter SL Mannelqvist M Dutt A Getz G Stefansson IM. Integrated Genomic Profiling of Endometrial Carcinoma Associates Aggressive Tumors With Indicators of PI3 Kinase Activation. Proc Natl Acad Sci USA (2009) 106:4834–9. 10.1073/pnas.0806514106 PMC2660768 19261849

[25] Guo H Kong W Zhang L Han J Clark LH Yin Y. Reversal of Obesity-Driven Aggressiveness of Endometrial Cancer by Metformin. Am J Cancer Res (2019) 9:2170–93. PMC6834476 31720081

[26] Cantrell LA Zhou C Mendivil A Malloy KM Gehrig PA Bae-Jump VL. Metformin Is a Potent Inhibitor of Endometrial Cancer Cell Proliferation–Implications for a Novel Treatment Strategy. Gynecol Oncol (2010) 116:92–8. 10.1016/j.ygyno.2009.09.024 PMC2789879 19822355

[27] Bae-Jump V Sill M Gehrig PA Moxley K Hagemann AR Waggoner SE. A Randomized Phase II/III Study of Paclitaxel/Carboplatin/Metformin Versus Paclitaxel/Carboplatin/Placebo as Initial Therapy for Measurable Stage III or IVA, Stage IVB, or Recurrent Endometrial Cancer: An NRG Oncology/GOG Study. 51st Annual Meeting of the Society of Gynecologic Oncology, April 2020, Virtual Meeting Due to COVID-19. Gynecol Oncol (2020) 159(Supplement 1):7 10.1016/j.ygyno.2020.06.013 PMC12368636 40056832

[28] Graham GG Punt J Arora M Day RO Doogue MP Duong JK. Clinical Pharmacokinetics of Metformin. Clin Pharmacokinet (2011) 50:81–98. 10.2165/11534750-000000000-00000 21241070

[29] Aoki M Terada T Kajiwara M Ogasawara K Ikai I Ogawa O. Kidney-Specific Expression of Human Organic Cation Transporter 2 (OCT2/SLC22A2) is Regulated by DNA Methylation. Am J Physiol Renal Physiol (2008) 295:F165–70. 10.1152/ajprenal.90257.2008 18508876

[30] Scheen AJ. Clinical Pharmacokinetics of Metformin. Clin Pharmacokinet (1996) 30:359–71. 10.2165/00003088-199630050-00003 8743335

[31] Choudhary GS Al-Harbi S Almasan A. Caspase-3 Activation Is a Critical Determinant of Genotoxic Stress-Induced Apoptosis. Methods Mol Biol (2015) 1219:1–9. 10.1007/978-1-4939-1661-0_1 25308257

[32] Perry SW Norman JP Barbieri J Brown EB Gelbard HA. Mitochondrial Membrane Potential Probes and the Proton Gradient: A Practical Usage Guide. Biotechniques (2011) 50:98–115. 10.2144/000113610 21486251 PMC3115691

[33] Lee WK Reichold M Edemir B Ciarimboli G Warth R Koepsell H. Organic Cation Transporters OCT1, 2, and 3 Mediate High-Affinity Transport of the Mutagenic Vital Dye Ethidium in the Kidney Proximal Tubule. Am J Physiol Renal Physiol (2009) 296:F1504–13. 10.1152/ajprenal.90754.2008 19357179

[34] Lim S Kaldis P. Cdks, Cyclins and CKIs: Roles Beyond Cell Cycle Regulation. Development (2013) 140:3079–93. 10.1242/dev.091744 23861057

[35] Grossmann ME Yang DQ Guo Z Potter DA Cleary MP. Metformin Treatment for the Prevention and/or Treatment of Breast/Mammary Tumorigenesis. Curr Pharmacol Rep (2015) 1:312–23. 10.1007/s40495-015-0032-z PMC4577062 26405648

[36] Dowling RJ Lam S Bassi C Mouaaz S Aman A Kiyota T. Metformin Pharmacokinetics in Mouse Tumors: Implications for Human Therapy. Cell Metab (2016) 23:567–8. 10.1016/j.cmet.2016.03.006 27076069

[37] Fatehi Hassanabad A MacQueen KT. Molecular Mechanisms Underlining the Role of Metformin as a Therapeutic Agent in Lung Cancer. Cell Oncol (Dordr) (2021) 44:1–18. 10.1007/s13402-020-00570-0 33125630 PMC12980714

[38] Jin DH Kim Y Lee BB Han J Kim HK Shim YM. Metformin Induces Cell Cycle Arrest at the G1 Phase Through E2F8 Suppression in Lung Cancer Cells. Oncotarget (2017) 8:101509–19. 10.18632/oncotarget.21552 PMC5731892 29254182

[39] Takahashi A Kimura F Yamanaka A Takebayashi A Kita N Takahashi K. Metformin Impairs Growth of Endometrial Cancer Cells *via* Cell Cycle Arrest and Concomitant Autophagy and Apoptosis. Cancer Cell Int (2014) 14:53. 10.1186/1475-2867-14-53 24966801 PMC4070401

[40] Safe S Nair V Karki K. Metformin-Induced Anticancer Activities: Recent Insights. Biol Chem (2018) 399:321–35. 10.1515/hsz-2017-0271 29272251

[41] Buzzai M Jones RG Amaravadi RK Lum JJ DeBerardinis RJ Zhao F. Systemic Treatment With the Antidiabetic Drug Metformin Selectively Impairs P53-Deficient Tumor Cell Growth. Cancer Res (2007) 67:6745–52. 10.1158/0008-5472.CAN-06-4447 17638885

[42] Hsieh Li SM Liu ST Chang YL Ho CL Huang SM. Metformin Causes Cancer Cell Death Through Downregulation of P53-Dependent Differentiated Embryo Chondrocyte 1. J BioMed Sci (2018) 25:81. 10.1186/s12929-018-0478-5 30442142 PMC6238313

[43] Inoki K Zhu T Guan KL. TSC2 Mediates Cellular Energy Response to Control Cell Growth and Survival. Cell (2003) 115:577–90. 10.1016/S0092-8674(03)00929-2 14651849

[44] Arner P Kulyte A Batchelor K Laurencikiene J Livingston J Ryden M. Mapping of Biguanide Transporters in Human Fat Cells and Their Impact on Lipolysis. Diabetes Obes Metab (2018) 20:2416–25. 10.1111/dom.13395 PMC6133731 29862627

[45] Zakikhani M Dowling RJ Sonenberg N Pollak MN. The Effects of Adiponectin and Metformin on Prostate and Colon Neoplasia Involve Activation of AMP-Activated Protein Kinase. Cancer Prev Res (Phila) (2008) 1:369–75. 10.1158/1940-6207.CAPR-08-0081 19138981

[46] Di Matteo S Nevi L Overi D Landolina N Faccioli J Giulitti F. Metformin Exerts Anti-Cancerogenic Effects and Reverses Epithelial-to-Mesenchymal Transition Trait in Primary Human Intrahepatic Cholangiocarcinoma Cells. Sci Rep (2021) 11:2557. 10.1038/s41598-021-81172-0 33510179 PMC7844056

[47] Hampsch RA Wells JD Traphagen NA McCleery CF Fields JL Shee K. AMPK Activation by Metformin Promotes Survival of Dormant ER(+) Breast Cancer Cells. Clin Cancer Res (2020) 26:3707–19. 10.1158/1078-0432.CCR-20-0269 PMC7367755 32321715

[48] Faria J Negalha G Azevedo A Martel F. Metformin and Breast Cancer: Molecular Targets. J Mammary Gland Biol Neoplasia (2019) 24:111–23. 10.1007/s10911-019-09429-z 30903363

[49] Leskela S Perez-Mies B Rosa-Rosa JM Cristobal E Biscuola M Palacios-Berraquero ML. Molecular Basis of Tumor Heterogeneity in Endometrial Carcinosarcoma. Cancers (Basel) (2019) 11(7):964. 10.3390/cancers11070964 PMC6678708 31324031

[50] Podsypanina K Lee RT Politis C Hennessy I Crane A Puc J. An Inhibitor of mTOR Reduces Neoplasia and Normalizes P70/S6 Kinase Activity in Pten+/- Mice. Proc Natl Acad Sci USA (2001) 98:10320–5. 10.1073/pnas.171060098 PMC56959 11504907

[51] Grunwald V DeGraffenried L Russel D Friedrichs WE Ray RB Hidalgo M. Inhibitors of Mtor Reverse Doxorubicin Resistance Conferred by PTEN Status in Prostate Cancer Cells. Cancer Res (2002) 62:6141–5. 12414639

[52] Neshat MS Mellinghoff IK Tran C Stiles B Thomas G Petersen R. Enhanced Sensitivity of PTEN-Deficient Tumors to Inhibition of FRAP/mTOR. Proc Natl Acad Sci USA (2001) 98:10314–9. 10.1073/pnas.171076798 PMC56958 11504908

[53] Calle EE Rodriguez C Walker-Thurmond K Thun MJ. Overweight, Obesity, and Mortality From Cancer in a Prospectively Studied Cohort of U. S. adults. New Engl J Med (2003) 348:1625–38. 10.1056/NEJMoa021423 12711737

[54] Lu KH Broaddus RR. Endometrial Cancer. N Engl J Med (2020) 383:2053–64. 10.1056/NEJMra1514010 33207095

[55] Schuler KM Rambally BS DiFurio MJ Sampey BP Gehrig PA Makowski L. Antiproliferative and Metabolic Effects of Metformin in a Preoperative Window Clinical Trial for Endometrial Cancer. Cancer Med (2015) 4:161–73. 10.1002/cam4.353 PMC4329001 25417601

