# Supplementary figures and images for: The Effects of NT-1044, a Novel AMPK Activator, on Endometrial Cancer Cell Proliferation, Apoptosis, Cell Stress and In Vivo Tumor Growth
Source: Front Oncol. 2021 Aug 5;11:690435. doi: 10.3389/fonc.2021.690435 (PMC8377676; doi:10.3389/fonc.2021.690435)

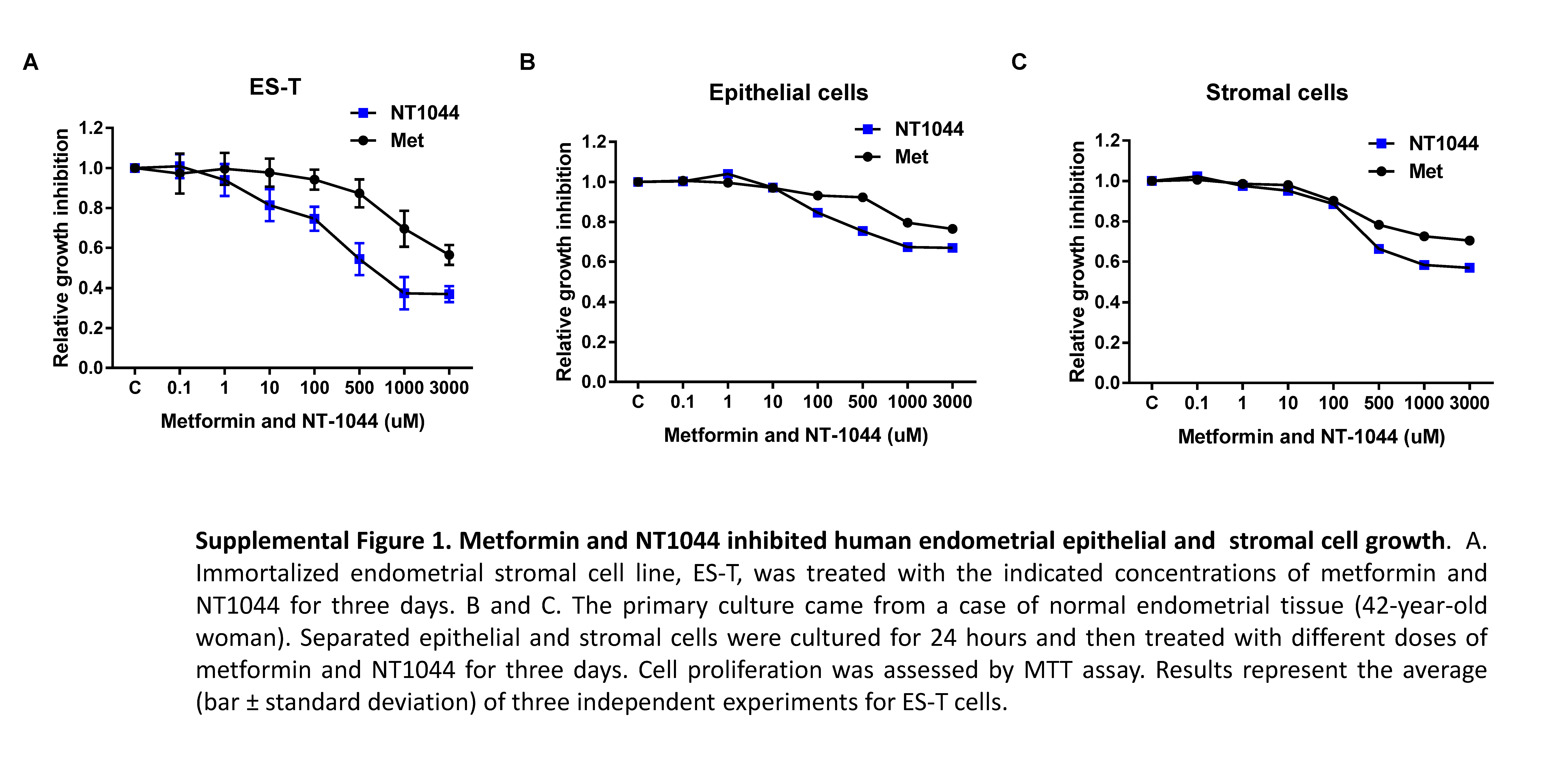

Supplement: Supplementary file 1 [file Image_1.tif]

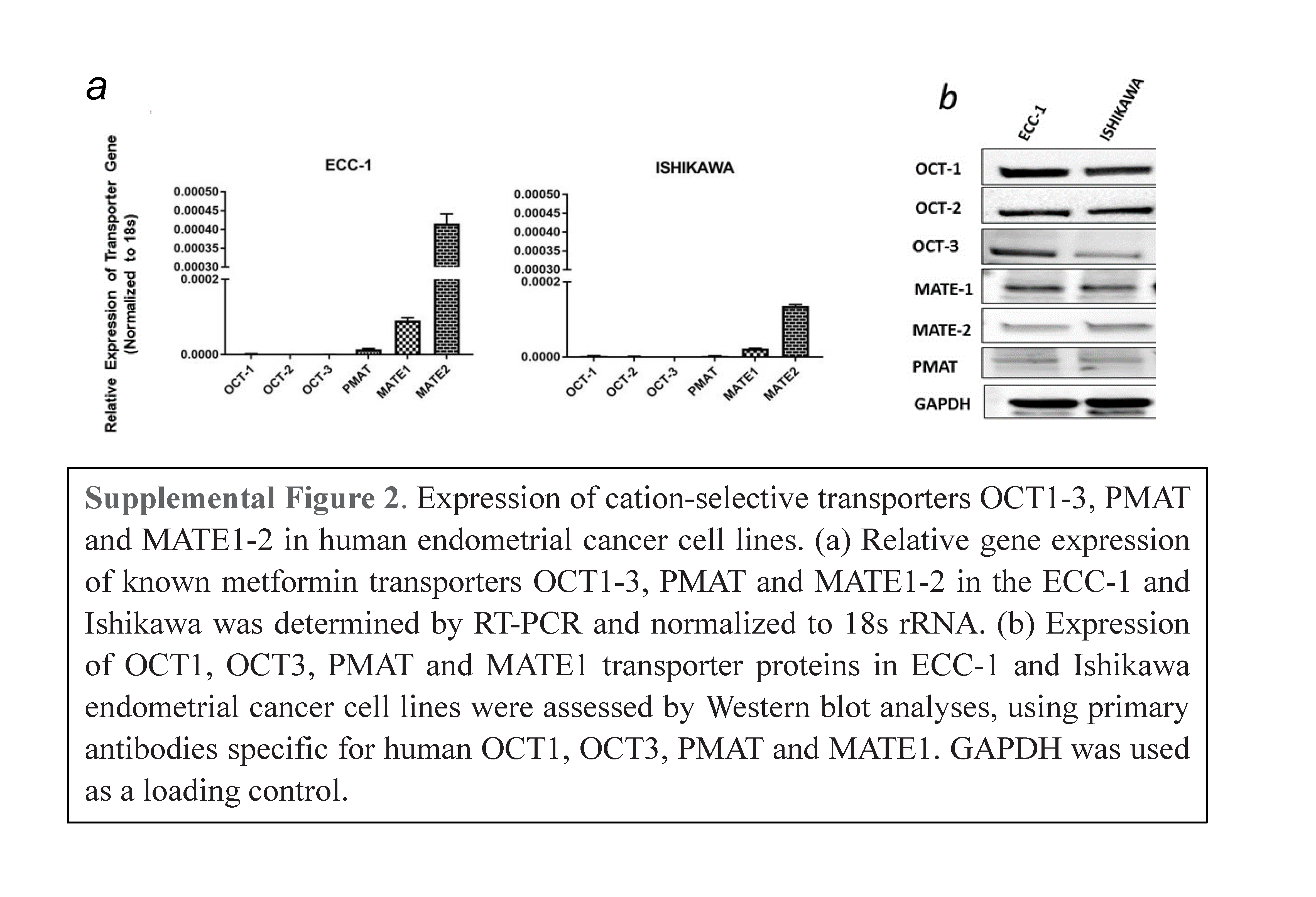

Supplement: Supplementary file 2 [file Image_2.tiff]
